# Originator versus biosimilar adalimumab in hidradenitis suppurativa: A multicenter real-world analysis of clinical response, maintenance of response, and switching

**DOI:** 10.1016/j.jdin.2025.05.023

**Published:** 2025-10-01

**Authors:** Marra Aghajani, James Pham, Tara Sholji, Kate Burrell, Cindy Kok, John W. Frew

**Affiliations:** a The Skin Hospital, Sydney, Australia; b University of New South Wales, Sydney, Australia

**Keywords:** adalimumab, biosimilar, hidradenitis suppurativa, survival analysis

## Abstract

**Background:**

Adalimumab is a mainstay treatment for hidradenitis suppurativa (HS); however, high-costs limit accessibility. While biosimilars offer an affordable alternative, concerns remain regarding clinical equivalence and the impact of switching.

**Objective:**

To evaluate the clinical response of originator versus biosimilar adalimumab in HS and assess outcomes following nonmedical switching.

**Methods:**

This multicenter, real-world study included treatment naïve HS patients initiated on originator or biosimilar adalimumab between April 2021 and December 2024. Outcomes were compared between patients remaining on originator and those switched to biosimilar after 16 weeks. Clinical responses were assessed using Hidradenitis Suppurativa Clinical Response 50, Hidradenitis Suppurativa Clinical Response 75, and International Hidradenitis Suppurativa Severity Scoring System 55, with time-to-event analyses evaluating loss of clinical response.

**Results:**

Patients on originator adalimumab (*n* = 186) demonstrated greater proportions of clinical response than biosimilars (*n* = 127), with time-to-event analysis showing a significant difference between curves (100 vs 52 weeks; hazard ratio = 2.73; *P* < .0001). Patients switched to biosimilars (*n* = 71) and experienced higher rates of loss of response than those maintained on originator (*n* = 71) (50 vs 87 weeks; hazard ratio = 2.42; *P* = .0003).

**Limitations:**

Limitations include its retrospective design and variable biosimilar formulations.

**Conclusion:**

Significant differences in clinical response, loss of response, and time-to-event analysis between originator and biosimilar adalimumab in HS underscore the need for consistent treatment and robust monitoring to optimize management.

**Capsule Summary**

• Biosimilar adalimumab has improved accessibility in treating hidradenitis suppurativa; however, real-world data on its clinical equivalence are scant.
• A significant difference in the proportion of patients achieving and losing clinical response was observed between originator and biosimilar adalimumab. Clinicians should be aware of emerging evidence suggesting nonequivalency.

## Introduction

Hidradenitis suppurativa (HS) is a chronic inflammatory skin condition characterized by recurrent painful nodules, abscesses, tunnels, and scarring in apocrine gland-bearing regions.^1^ Biologics including adalimumab are the mainstay of therapy in moderate to severe disease alongside surgery,^2^ but access is limited by the high cost of these medications. Biosimilars are biotherapeutic products designed to replicate the efficacy, safety, and quality of the original biologic while being more affordable to both patients and health care systems.^3^ Formal phase 3 studies for biosimilar adalimumab in HS have not been conducted due to presumed equivalency based on studies in psoriasis.^4^ Case series and cohort studies have illustrated a Hidradenitis Suppurativa Clinical Response (HiSCR) 50 response rate of 40% to 50% for biosimilar adalimumab in bionaive individuals.^5^^,^^6^

Despite regulatory approval processes designed to ensure comparability to the originator biologic, biosimilars are not identical replicas due to the complexity of manufacturing processes involving living cells.^7^ Emerging evidence suggests that switching between originator and biosimilar biologics may lead to loss of response, adverse events, and reduced patient adherence.^8^, ^9^, ^10^ Additionally, the potential for antidrug antibody development is a key consideration when evaluating originator adalimumab to its biosimilars, as even minor variations in manufacturing processes or formulation can impact immunogenicity and compromise treatment efficacy.^11^ In the context of HS, where biologic treatment options are limited, further research is essential to confirm that biosimilars consistently deliver reliable outcomes for patients.

This multicenter real-world analysis evaluated the differences in achieving clinical response, loss of response, and time-to-event outcomes in treatment naïve HS patients commenced on either originator or biosimilar adalimumab. Additionally, the impact of nonmedical switching from originator to biosimilar adalimumab on patient outcomes was examined.

## Methods

This study examined clinical response data from 3 HS clinics for patients treated between April 2021 and December 2024. Two distinct cohorts were identified for analysis in this study. The first cohort comprised of bio naïve patients who were commenced on either adalimumab originator or biosimilar during the period of interest (Bio Naïve Cohort). The second cohort identified patients who, after commencing on originator adalimumab for the first 16 weeks of therapy, achieved clinical response and were subsequently transitioned over to biosimilar adalimumab (Switched Cohort) due to changing formulary requirements at our institutions. The institutions involved were in the process of replacing originator adalimumab with biosimilar adalimumab for cost-saving purposes, although the cost of both agents to patients (originator and biosimilar agents) were the same given the nature of the socialized health care system in Australia. This cohort was matched by age, gender, body mass index (BMI), and smoking status to individuals who initiated and remained on originator therapy.

All patients were subject to the same eligibility criteria for their data to be included in analysis. Patients were included if they had a formal diagnosis of HS by a dermatologist, were prescribed adalimumab (either originator or biosimilar during the period examined), and were bio naïve at the time of adalimumab (originator or biosimilar) commencement. All other therapies (topical, hormonal, etc.) were allowed to continue as prescribed. Patients were excluded if they were pregnant or breastfeeding during the period examined. All patients adhered to the same Federal Drug Administration/European Medicines Agency/Therapeutic Goods Administration dosing regimen (160 mg at week 0, 80 mg every 2 weeks thereafter). No patients were on any additional immunosuppressive medications aimed at reducing antidrug antibody formation.

The analysis consisted of demographic and disease characteristics, with differences compared using mean for normally distributed variables and medians for non-normally distributed variables. Clinical response was measured using HiSCR-50, HiSCR-75, and International Hidradenitis Suppurativa Severity Scoring System (IHS4) 55. Time-to-event analysis was employed to analyze time to loss of clinical response as measured by the loss of HiSCR-50 in both the originator and biosimilar cohorts. Regression analysis was used to examine disease and demographic factors that may be associated with maintenance or loss of clinical response in each cohort. Sub-analysis was undertaken regarding patients who had commenced originator adalimumab, demonstrated a clinical response, and were then switched to a biosimilar medication after week 16 of therapy. This cohort was compared to an age, gender, Hurley stage, smoking status, and BMI-matched cohort of patients continuing originator adalimumab.

## Results

### Bio naïve cohort

A total of 313 individual patients with dermatologist-diagnosed HS who met the edibility criteria were identified for the bio naïve cohort. Demographic and disease characteristics are presented in Table I. No significant differences were identified between originator and biosimilar cohorts.

**Table I:** Demographics and disease characteristics of bio naïve patients included in this analysis

Bio naïve cohort	Originator (*n* = 186)	Biosimilar (*n* = 127)	*P* value
Age (median, IQR)	31 (24-42)	30 (24-39)	.33
Sex (male/female)	91/95	62/64	.39
Hurley stage (stage 2/3)	129/57	93/33	.54
Smoking status	94 (50.5%)	59 (46.4%)	.76
BMI	31.5 (26-37)	29 (27-36)	.27
Length of time with disease (y) (median, IQR)	11 (6-14)	11 (7-14)	.99
Achieving HiSCR-50 at wk 16	118 (63.4%)	72 (57%)	.27
Achieving HiSCR-50 at wk 52	**95 (51%)**	**30 (24%)**	**.0001**
Achieving HiSCR-75 at wk 16	**61 (33%)**	**38 (30%)**	**.008**
Achieving HiSCR-75 at wk 52	**45 (24%)**	**18 (14%)**	**.0076**
Achieving IHS4-55 at wk 16	113 (61%)	70 (55%)	.26
Achieving IHS4-55 at wk 52	**83 (45%)**	**28 (22%)**	**.0001**

Values in bold represent statistically significant.

*BMI*, Body mass index; *HiSCR*, Hidradenitis Suppurativa Clinical Response; *IHS4*, International Hidradenitis Suppurativa Severity Scoring System; *IQR*, interquartile range.

**Fig 1:**
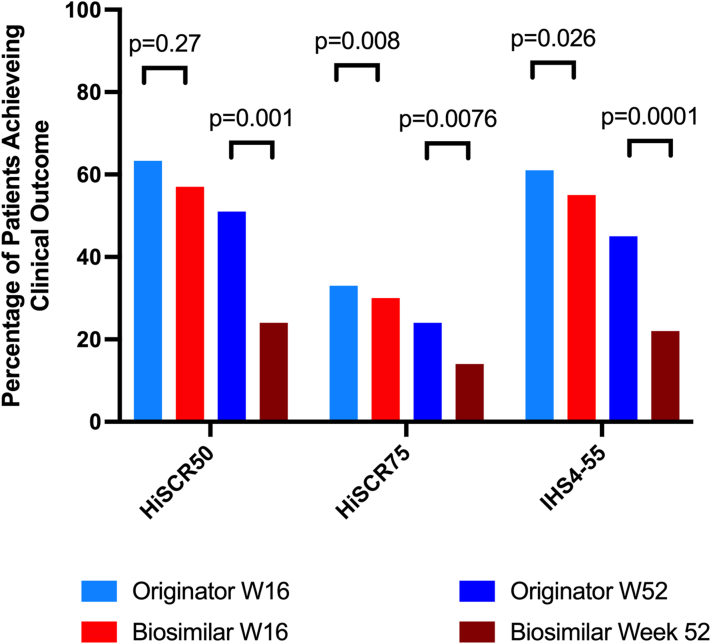
Hidradenitis suppurativa. Proportion of patients achieving HiSCR-50, HiSCR-75, and IHS4-55 outcome measures at week 16 and week 52 in the bio naïve cohort (*n* = 313). *HiSCR*, Hidradenitis Suppurativa Clinical Response; *IHS4*, International Hidradenitis Suppurativa Severity Scoring System.

At week 16, clinical responses measured by HiSCR-50, HiSCR-75, and IHS4-55 criteria were compared between patients receiving originator adalimumab and those on biosimilar adalimumab (Fig 1). The proportion of patients achieving HiSCR-50 was similar between the groups (*P* = .27), while a significantly higher percentage of patients in the originator group achieved HiSCR-75 compared to the biosimilar group (*P* = .008). For IHS4-55, no statistically significant difference was observed between the groups (*P* = .26). By week 52, the originator group demonstrated consistently superior outcomes across all clinical measures, with significantly higher percentages of patients achieving HiSCR-50 (*P* = .0001), HiSCR-75 (*P* = .0076), and IHS4-55 (*P* = .0001) compared to the biosimilar group.

**Fig 2:**
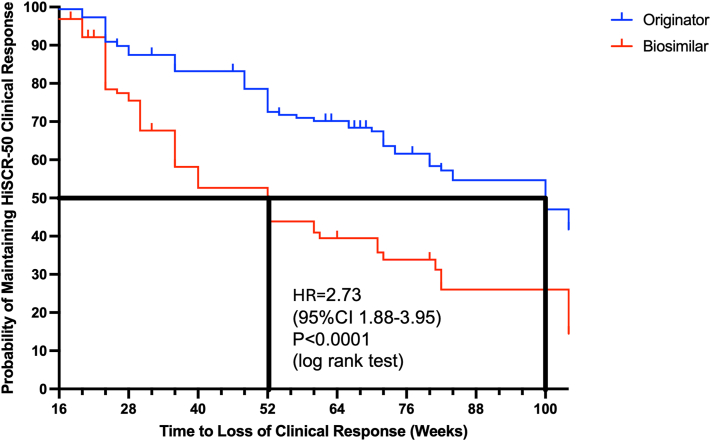
Hidradenitis suppurativa. Time-to-event analysis and comparing originator adalimumab (*n* = 186) to biosimilar adalimumab (*n* = 127) in bio naïve individuals.

Time-to-event analysis for the bio naïve cohort demonstrated a significant difference between curves with the log rank test (*P* < .0001). The median time to loss of response for originator adalimumab was 100 weeks compared to 52 weeks for biosimilar adalimumab (Fig 2). This was equivalent to a hazard ratio of 2.73 (1.88-3.95) for having a loss of response to biosimilar adalimumab than originator adalimumab in this cohort at any time during the first 104 weeks of therapy.

Regression analysis of the bio naïve cohort demonstrated Hurley stage, BMI, and biosimilar use as significant covariates in the odds of loss of clinical response. Biosimilar use was associated with a 77% increase in the odds of loss of clinical response compared to originator adalimumab (odds ratio = 1.767; 95% confidence interval: 1.063 to 2.960; *P* = .03) after accounting for BMI and Hurley stage (Table II).

**Table II:** Regression analysis for secondary loss of clinical response from the bio naïve cohort

Variable	Odds ratio	95% CI (profile likelihood)	*P* value
Intercept	0.002202	0.0001494-0.02792	<.0001
Age	1.037	0.915-1.060	.1010
Sex	0.6665	0.3922-1.130	.1321
Hurley stage	**3.846**	**1.675-9.245**	**.0019**
BMI	**1.051**	**1.010-1.096**	**.0161**
Tunnels	1.052	0.4595-2.316	.9006
Smoking status	1.081	0.6148-1.890	.7859
Biosimilar adalimumab	**1.767**	**1.063-2.960**	**.0290**

Values in bold represent statistically significant.

*BMI*, Body mass index; *CI*, confidence interval.

### Biosimilar switching cohort

A total of 71 individuals treated with originator adalimumab who achieved HiSCR-50 at week 16 and were subsequently switched to biosimilar adalimumab were identified. 71 matched individuals who remained on the adalimumab originator were included for comparison. No significant differences in age, sex, BMI, Hurley stage, or smoking status were identified between the 2 cohorts (Supplementary Table I, available via Mendeley at 10.17632/jx9gfzyrnw.1). Details of the range of biosimilar products used are presented in Supplementary Figs 1 and 2, available via Mendeley at 10.17632/jx9gfzyrnw.1.

**Fig 3:**
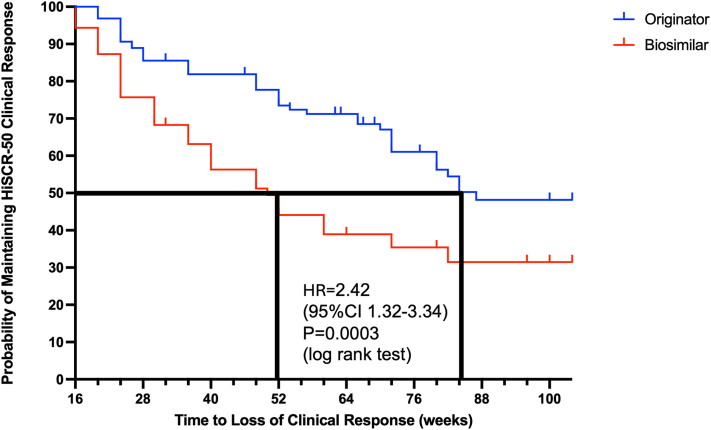
Hidradenitis suppurativa. Time-to-event analysis of time to loss of HiSCR-50 response after switch from originator to biosimilar adalimumab in week 16 clinical responders. *HiSCR*, Hidradenitis Suppurativa Clinical Response.

Time-to-event analysis demonstrated significant differences between curves using the log rank test between the 2 cohorts (*P* = .0003). The median time to loss of response for originator adalimumab was 87 weeks compared to 50 weeks for biosimilar adalimumab (Fig 3). This was equivalent to a hazard ratio of 2.42 (1.32-3.34) for loss of clinical response after switching from originator to biosimilar adalimumab at any timepoint during the period examined.

Regression analysis demonstrated BMI, Hurley stage, and biosimilar use as significant covariates in loss of clinical response (Table III). Switch to biosimilar adalimumab was associated with a 181% increase in the odds of loss of clinical response compared to originator adalimumab (odds ratio = 2.807; 95% confidence interval: 1.633 to 3.399; *P* = .02) after accounting for Hurley stage and BMI (Table III).

**Table III:** Regression analysis for loss of clinical response in switched cohort

Variable	Odds ratio	95% CI (profile likelihood)	*P* value
Intercept	0.003891	0.0002903-0.04569	<.0002
Age	1.004	0.815-1.143	.465
Sex	0.798	0.674-1.034	.6034
Hurley stage	**2.658**	**1.398-4.621**	**.0019**
BMI	**1.434**	**1.119-1.669**	**.0161**
Tunnels	1.129	0.5598-1.923	.6904
Smoking status	1.887	0.4658-1.993	.6588
Biosimilar adalimumab	**2.807**	**1.633-3.399**	**.0024**

Values in bold represent statistically significant.

*BMI*, Body mass index; *CI*, confidence interval.

## Discussion

This study revealed that bio naïve patients commenced on originator adalimumab achieved greater proportions of clinical response compared to biosimilar adalimumab. The median time to loss of response for originator adalimumab was 100 weeks versus 52 weeks for biosimilar adalimumab, with biosimilar use associated with a 77% increase in the odds of loss of clinical response compared to those commenced on originator adalimumab. Moreover, patients transitioned to biosimilar adalimumab experienced a median time to loss of response of 50 weeks compared to 87 weeks in those who remained on the originator. The switched cohort had a 181% increase in the odds of losing clinical response, even after adjusting for Hurley stage and BMI.

The existing literature presents mixed perspectives on the efficacy and safety of biosimilars compared to originator biologics in HS. Ricceri et al reported that all 11 patients in their cohort treated with biosimilars achieved HiSCR, although these outcomes were accompanied with higher rates of adverse events, particularly injection site pain.^12^ Similarly, Patil et al described promising HiSCR rates in 2 cases of HS treated with biosimilars, but the concurrent use of methotrexate in these cases raised concerns about the standalone efficacy of biosimilars.^13^ While these studies highlight the potential utility of biosimilars in treatment naïve patients, their findings are limited by small sample sizes and short follow-up periods. In contrast, a multicenter study involving 326 HS patients identified superior treatment efficacy with originator adalimumab (82.2%) compared to biosimilars (60.5%) after 10 months of treatment.^14^ These results are consistent with our findings, reinforcing the observation that treatment naïve patients initiated on originator adalimumab are more likely to achieve a durable clinical response.

Loss of treatment efficacy and increased adverse events have been reported in up to 58.8% of HS patients following nonmedical switching from originator biologics to biosimilars.^9^^,^^10^ Side effects, particularly injection site pain, significantly reduced tolerability and contributed to high discontinuation rates, with up to 43.2% of patients ceasing biosimilar therapy after switching.^8^ While switching back to the originator resolved most injection site reactions, a third of patients failed to regain their initial clinical response, suggesting potential irreversible immunological changes.^10^ Our findings corroborate these concerns, demonstrating a significantly shorter median time to loss of response in patients who switched to biosimilars compared to those who remained on the originator. These results underscore the need for careful consideration of nonmedical switching and highlight the importance of treatment consistency to optimize long-term outcomes in stable HS patients.

This study’s strengths include its multicentric design and use of real-world patient data, enhancing generalizability. The matched analysis of switch and nonswitch cohorts provided a robust framework for comparing clinical outcomes. Known confounders such as Hurley stage, BMI, and presence of tunnels have been identified and accounted for in this analysis, ensuring the robustness of our conclusions. The sample size of our study is a limitation which may be overcome in future studies with prospective designs with larger, more diverse populations and extended follow-up periods.

## Conclusion

HS patients initiated on biosimilar adalimumab, or those transitioned from the originator to a biosimilar, experienced lower rates of clinical response, higher rates of loss of clinical response, and shorter times to loss of response. These findings align with existing evidence suggesting that nonmedical switching may compromise treatment efficacy and underscores the need for careful evaluation and the personalized use of biosimilars in clinical practice. Additionally, these results highlight the need for equivalence studies in HS and not purely adopting the equivalency results from other conditions such as psoriasis. Future studies should identify and refine strategies to address the potential challenges associated with treatment switching. These efforts will be critical given the emerging markets of biosimilars for interleukin (IL) 12/IL-23 and IL-17 agents.

## Conflicts of interest

Dr Frew has conducted advisory work for Janssen, Boehringer-Ingelheim, Pfizer, Kyowa Kirin, LEO Pharma, Regeneron, Chemocentryx, Abbvie, and UCB; participated in trials for Pfizer, UCB, Boehringer-Ingelheim, Eli Lilly, and CSL; and received research support from Ortho Dermatologics, Sun Pharma, and La Roche Posay. J.W.F. is the co-Editor-in-Chief of the Australasian Journal of Dermatology and coauthor of this article. Drs Aghajani, Pham, and Sholji; Burrell; and Dr Kok have no conflicts of interest to declare.
